# The Conference of the Birds: An Old Artistic Concept Making Sense in Modern Sciences

**DOI:** 10.32598/bcn.9.4.297

**Published:** 2018-07-01

**Authors:** Mohammad Reza Saebipour, Marzieh Zare, Kazem Ghaemi, Mohammad Taghi Joghataie

**Affiliations:** 1. Department of Anatomy, School of Medicine, Birjand University of Medical Sciences, Birjand, Iran.; 2. School of Computer Sciences, Institute for Research in Fundamental Sciences, Tehran, Iran.; 3. Department of Neurosurgery, School of Medicine, Birjand University of Medical Sciences, Birjand, Iran.; 4. Department of Anatomy, School of Medicine, Iran University of Medical Sciences, Tehran, Iran.; 5. Cellular and Molecular Research Center, Iran University of Medical Sciences, Tehran, Iran.

**Keywords:** Conference of birds, Art, Science, Brain, Mind

## Abstract

In this article, we will discuss scientific aspects of an old Persian story, Simorgh, in the book of *The Conference of the Birds*. The story is fulfilled with artistic and philosophical metaphors that make sense in two hot topics of the contemporary modern sciences i.e. cognitive science and complexity science. The poet addresses some humanity’s bygone concerns and fundamental questions about self, the quality that shapes a person’s uniqueness, and essential existence. The sophisticated language used in the poem contains allusions, symbols, and implications that are interpreted in five main topics. We think that the story deserves to be the touchstone for questions on the nature of the mind, including the profound question of humanity’s search for self and meaning of life.

## Highlights

- “The Conference of Birds,” a Persian old story, was discussed from the modern scientific perspective.
- The story narrates the journey of a group of birds toward the living place of a legendary bird, Simorgh, as a symbol of the human search for self.
- The characteristics of the story are interpreted as significant aspects of the brain as a complex system.

## Plain Language Summary

Two old humankind mysteries; consciousness and meaning of life have been addressed concurrently since thousands of years ago in ancient Persian culture. We propose that Attar suggested an artistic and philosophical solution for these two questions “The Conference of Birds,” has meaningful scientific implications. Collective behavior of neurons which leads to consciousness and also the collective behavior of humanity which composes our civilization are mechanistically similar to swarm intelligence.

## 1. Introduction

Today, scientists endeavor to understand the mind and the brain more than ever in the history of science. Many mega-projects such as Connectome, BRAIN, Blue Brain, etc. have been funded to study animal brains in different coarse-grained scales, from the microscopic (i.e. relationship between neurons) to the macroscopic (i.e. behavior and consciousness) level. Cognitive science, as a multidisciplinary insight including different disciplines (neuroscience, psychology, linguistics, anthropology, philosophy and artificial intelligence), studies the brain and the mind (Thagard, 2008; Miller, 2003).

One of the foremost issues in science in general, and in cognitive science in particular, is to understand the concept of self, i.e. the quality that shapes a person’s uniqueness or essential existence. The question has engaged many philosophers, scientists, and neurophilosophers as much as poets and artists. The self is regarded as the origin of consciousness, responsible for an individual’s ideas and activities, and the substantial nature of a soul, which sustains and unites consciousness over time.

One of the controversial debates is the relationship between the brain and the mind, and whether the brain is a computational system that functions as an input-output system. However, cognitive scientists believe that the brain is not a computer, and the complexity of its functions is far from the predictive processing of robots or intelligent devices.

The basis of the conflict refers to the two general perspectives in science, including reductionism and holism. Rene Descartes was the first to introduce the former (Webster & Doniger, 1999), based on the notion that understanding a system is possible by reducing it to its components. According to Descartes, the universe is an abstract machine, and the study of its components is sufficient to understand the whole system. In contrast, in a holistic framework, determination and explanation of a system’s behavior are not possible just by the reductionist perspective.

South African statesman, Jan Smuts, in his book *Holism and Evolution* (Smuts, 1927), proposed the term “holism” as a natural tendency to form wholes that are larger than the sum of the parts through creative progress. He denoted holism as the lost key of contemplation. Several systems have been studied with a holistic approach, such as socioculturalism, education, health care, economics, etc. In the mid-20^th^ century, holism surpassed to system thinking and its derivatives, like the sciences of chaos and complexity. A complex system exists when there is a collection of various components at different scales with intrinsic and inseparable parts, and the elimination of some higher parts in order to reduce the system to a lower size is impossible (Bar-Yam, 2002).

Various studies on collective behavior reveal emergent behavior as the result of single entities-forming the complex system-operating in an environment. System thinking, with multidisciplinary insight, enables the possibility of studying the brain as a system similar to a flock of birds, school of fish, etc. and to find the complex patterns corresponding to its behavior (Zare & Grigolini 2013; Vanni, Luković & Grigolini, 2011). Today, cognitive science and complexity science are two fields that can be joined together to address difficult problems such as understanding the brain and the mind.

In this paper, we analyze a symbolic story of a legendary Persian bird, Simorgh similar to the western Phoenix-comprising today’s scientific and philosophical concerns of cognitive science. Having a long lasting influence on Persian poetry, Abu Hamid bin Abu Bakr Ibrahim (c. 1145–1221), known as Farīd ud-Dīn Attār (the apothecary), was a Persian poet who wrote the story in the book entitled *The Conference of the Birds* (Nott, 2016; Darbandi & Davis, 1984).

Attār’s handling of symbolisms in a long poem of approximately 4500 lines is a masterpiece reflected through the large context of the story. Setting the aesthetic aspects of the story aside, it encompasses profound scientific ideas that have never been explored among scientific communities. Relying on these aspects, noteworthy are the notions of system thinking and complexity science in cognitive studies, as well as the state of system thinking and holism in ancient Persian history.

### 2. Significance of the Story

Jalāl ad-Dīn Muhammad Rūmī (c. 1207–1273), known as Mawlana Rumi, was a 13^th^-century Persian poet, legist, Islamic intellectual, theologian, and Sufi Gnostic. Rumi’s influence outreaches geographical borders and ethnical divisions. His poems have been translated into many of the world’s languages and rendered into various formats. He described Attār as his spirit (Arberry, 2001) and praised him in a poem *Attār has searched through the seven cities of love while we have hardly passed the first alley* (Nasr, 2012).

Rumi’s appreciation of Attār reveals the same orientation of attitudes among Persian thinkers and philosophers of that time. Therefore, perception and realization of Attār’s message in the story, by the creation of metaphors and seemingly simple symbols, is worth particular consideration; speculating about Attār’s allusions, consideration of humanity’s bygone concerns and fundamental questions-as mentioned by many other poets-is required. Rumi’s concern about the mind which is accompanied by the meaning of life is expressed by the following verses (Rumi & Barks, 1995):

*All day I contemplate it, and then at night, I verbalize it.*
*Where did I emanate from, and what am I supposed to be doing? I have no conception.*
*My soul emanates from elsewhere, I’m sure of that, and I intend to culminate up there.*
*Who verbalizes words with my mouth? Who looks out with my ocular perceivers? What is the soul?*
*I cannot stop asking.*
*If I could taste one sip of an answer,*
*I could liberate from this prison for drunks*
*(Ch. 1: The Tavern, p. 2)*

Despite its importance for world literature and study of religion, Attār’s storybook, *The Conference of the Birds*, was not entirely translated until the mid-20^th^ century. However, translator of the Rubaiyat of Omar Khayyam, Edward FitzGerald (Briggs, 1998), worked on this concise translation of what he entitled *The Bird Parliament* in 1857. It was published after his death (FitzGerald passed away in 1883), in *Letters and Literary Remains*, edited by William Aldis Wright, in 1889. A contextual narration of the story in delicate English was released by Packard Humanities Institute over the Internet. Notably, Hellmut Ritter remarkable study of Attār’s thoughts and his poetic persona in the book, *Das Meer der Seele: Mensch, Welt und Gott in den Geschichten des Farīd ud-Dīn Attār (1955)*, was originally written in German that later was translated into Persian and English.

In 1979, Jean-Claude Carrière and Peter Brook published a play that has adopted the poem into a play called *La Conference des Oiseaux (The Conference of the Birds)*. Brook exhibited the play on a tour around rural Africa before presenting two immensely successful productions to the audiences in Paris and New York City at La MaMa Experimental Theatre Club (La MaMa, E.T.C). Several western musical artists used the poem. In particular, David Holland’s album was written as a metaphor for his own insight.

## 3. Simorgh in Persian Literature

Simorgh is a mythical bird frequently mentioned in Persian ancient literature, comparable to the western Phoenix (Arabic Ghoghnoos). It is a long-lived bird cyclically reborn (Barnhart, 1995). Both birds are associated with the sun. While the Phoenix is born from the ashes of its precedents (symbolizing revitalization, continuation and eternal survival). Simorgh was mentioned in the book of Zoroastrian, Avesta for the first time; she is a sapient and wise bird, aware of surreptitious secrets, described as a bird with immense wings, nesting on a remedial tree called Vispobish carrying the seeds of all plants (Schmidt, 2002).

**Figure 1.**
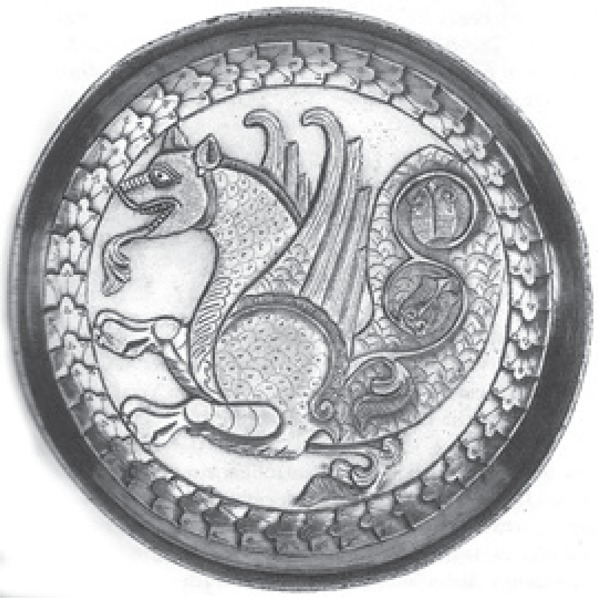
Simorgh sketched on coins during the kingdom of Yazdgerd III

Before Attār, Ferdowsi (c. 940–1020), the creator of the longest epic poem, *Shahnameh* (Book of Kings), (Lalani, 2010), described Simorgh’s involvement with prince Zal, wherein she is a tender-hearted, supportive and wise bird. Depictions of Simorgh are complicated, as seen in traces of Persian monuments where Simorgh is a bird with the peacock’s tail, an eagle’s body, dog’s head and a lion’s claws. The features of Simorgh were primarily sketched during the kingdom of Yazdgerd (III), the 38 and last king of the Sasanian Empire of Persia (c. 632–651) (Figure 1). In Azerbaijani and Kurdish folklore, Simorgh is the symbol of protection and potency. In Sufi poetry, Simorgh is used as a metaphor for God. However, Attār as a Sufi, created a new meaning for Simorgh in his poetic story, *The Conference of the Birds* (Darbandi & Davis, 1984).

## 4. The Story

Farīd ud-Dīn Attār depicts a captivating story of birds in the epic poem, *The Conference of the Birds* (Persian: *Mantiq-ut-Tair*). The poem starts with the conference of the all world birds to find the king. The wisest bird, Hoopoe, proposes that they should discover the legendary bird Simorgh. The group of birds starts the journey to cross seven valleys of quest, love, understanding, detachment, unity, astonishment, and finally deprivation and death, one after to find Simorgh.

Each bird is a moral symbol of human behavior and has an associated literacy purpose. The guiding bird is the hoopoe, while the nightingale symbolizes the lover. The parrot is searching for the origin of eternity, and the “fallen soul” who is in alliance with Satan is symbolized by peacock.

On the way to find the Simorgh, birds drop out of the journey one by one, claiming that they are not able to bear the journey or that the differences between them are too great to overcome. However, the wisest bird, Hoopoe, convinces them to continue the journey, advising them to focus on the integrity and ignore the conflicts between them. In the end, only thirty birds stay in the group as they reach Qaf, the dwelling place of Simorgh. It is worth mentioning that Simorgh [Si (thirty)+morgh (bird)] means “thirty birds” in Persian, referring to the number of birds that endured the journey. What happens at the end? All they discover is a water lake in which they see their own image and not the mythical Simorgh: What they were looking for exists within their collective self and in the totality of all things.

## 5. Interpretations and Implications of the Story

Simorgh, the story in the book *The Conference of the Birds* is opulent with artistic and philosophical implications, widely discussed in literature and philosophy. However, scientific aspects of the story make sense not only in sociology but also in brain sciences. In this article, we will highlight scientific implications of the story having been articulated in the symbolic and mysterious language of Attār. We will discuss similarities between elements of the story and scientific concepts of the brain in five main topics.

### 5.1. Cooperative Behavior and Synchrony

*The Conference of the Birds*, that aims to find the unknown, resembles collective cooperative behavior and swarm intelligence. It can be a metaphor of flocking as an example of a social complex system and the brain’s neuronal networks. Collective behavior among birds is elegantly expressed throughout the story, and in particular, at the final stage where the group of birds arrives at Qaf (the dwelling place of Simorg) and reaches unity within their collective selves. Attār depicts the concept of union in the reflectance of the birds’ image in the water lake as the legendary Simorgh.

The image also signifies that the meaning does not emerge from individuality but from cooperation and collective behavior among agents forming a society. The emergence of a unified moment and global order relies on the self-organization of scattered birds, each representing a corresponding human archetype. Interestingly this metaphor can be generalized to the brain level as many empirical studies have revealed the synergic behavior of several networks in the brain’s functions (Bressler, 1996; Salinas & Sejnowski, 2010) and different brain regions mediating different mental processes (Miller, 2003).

Hence, injuries to cortical and subcortical regions may lead to deficits in brain functions, depending on the affected region and neural networks that activate several sets of regions collaboratively working together toward the progression of a task. However, there is no evidence suggesting the existence of a central controller. Moreover, brain networks are not individually responsible for the emergence of consciousness, but the functional correlation and synchronization between them may lead to consciousness. The neurons tend to be companions in harmony, as was revealed in a beautiful study showing that neurons communication in the nervous system is comparable to friendship in social networks such as Facebook (Cossell et al., 2015).

**Figure 2.**
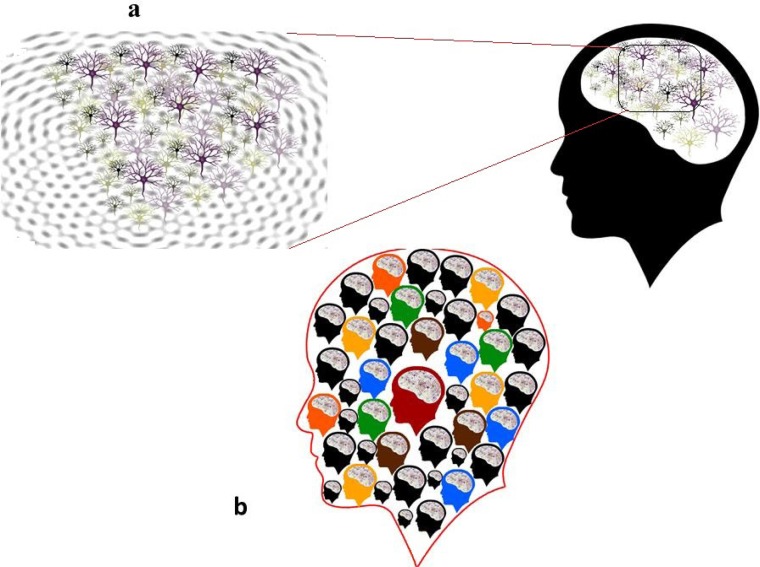
Neuron’s collective behavior resembling humans’ collective behavior (a) Ephaptic coupling adds a new dimension to the neuronal interactions in the brain and ultimately contributes to brain’s rhythmogenesis. This phenomenon is based on the sensitivity of each neuron to the activity of neighboring neurons and global brain activity. Propagation of the brain rhythms is crucial for emergence of consciousness. These social neurons (besides all other fascinating systems and neuronal networks) make the whole brain social by creating mirror neuron system (b) At the social level, each individual emphatic mind as an element of a complex system contributes to constructing a mega-mind of humanity.

Looking at neuronal behavior from another perspective, it is widely accepted that the major mechanism for signaling in a neural system is synaptic transmission and gap junctions (Song, Ampatzis, Björnfors, & El Manira, 2016). Recently ephaptic coupling has also come to the scene. When an electric field corresponding to the activity of one neuron polarizes the membranes of other neurons, an endogenous ephaptic transmission occurs in order to mediate propagation of self-regenerating neural waves (Faber, 2010). This endogenous ephaptic transmission or cell-by-volume coupling is responsible for rhythmogenesis, including Gamma and also hippocampal sharp wave-ripple (Figure 2a).

In particular, when we try to remember the context of a memory, wave-ripple coupling throughout the brain enforces the accessibility to our memory which represents an aspect of the most important theory of consciousness called global workspace (Tononi & Koch, 2015, Baars, 2005) (Figure 2a). Forty Hertz brain oscillations are linked to feature binding (Ross & Fujioka, 2016). In other words, the tendency of neurons to fire and wire together ultimately leads to the emergence of consciousness. Such social neurons tend to coordinate their behavior with the whole brain’s networks that constitute our social brain (Figure 2b).

Ephaptic coupling and gap junctions play a critical role in synchrony among neuronal ensembles. Synchronization occurs in many natural systems. Oscillators can spontaneously organize into a coherent, synchronized entity with a common frequency. In addition, synchronization between neurons is crucial in forming functional networks, and synchrony between brain regions modulates communication across large-scale brain networks. It has been suggested that binding by synchrony makes this communication possible (Engel, Fries, & Singer, 2001; Salinas & Sejnowski, 2000). Synchronized assemblies of neurons underlie cognitive functions such as recognition, recall, perception and attention. Most mental disorders are associated with abnormal synchronization (Uhlhaas & Singer, 2006).

Synchronized rhythms can enhance neural signals and modulate which brain networks “talk” to one another (Friston & Firth, 2015). Synchronous behavior also helps describe the boundaries between the self and the others, while concurrently allowing for compelling navigation of those boundaries in promoting effective interpersonal coordination so as to eliminate the boundaries between self and others (Friston & Firth, 2015).

### 5.2. Unity despite diversity

Birds naturally swarm with their congener while here, Attār emphasizes on variety and diversity of the society established by birds in the story making it distinct from other stories. Diversity, in fact, constructs system level robustness, granting for multiple responses to internal adaptations and external shocks (Page, 2010). In the story, Attār points out different birds to depict a diverse society, each sketching different traits of a complex system. Complex organisms must yield and retain a phenomenal diversity of cell and tissue types with a finite number of genes and molecular pathways (Jukam et al., 2013).

Similarly, it is very exciting how different and incompatible neurotransmitter systems have reached to this level of tranquil interactive and cooperative organization during the course of evolution. Brain strategy to sustain the balance between excitation and inhibition in a standpoint, in order to intercede precise administered message passing within and across hierarchical levels (Humphries, Wood, & Gurney, 2009).

Different brain rhythms also interact and behave harmonically and hierarchically. According to a recent research, neural oscillations at different frequencies are associated with a wide range of basic and higher cognitive processes in order to maintain information in working memory (Roux & Uhlhaas, 2014). In another study (Salazar, Dotson, Bressler, & Gray, 2012), across the frontoparietal network during visual working memory, task-dependent and content-specific synchronization of activity was revealed. It is unclear how properties of neuronal response is related to strength of connection. In this regard, Cossell et al. studied how functional organization of excitatory synaptic strength operates in the primary visual cortex (Cossell et al., 2015).

According to their results, complex organization of excitatory connection strengths mirrors the likeness of neuronal responses and suggests that scarce, strong connections modulate stimulus-specific response amplification in cortical microcircuits. In sum, despite the difference in features and functions of agents constructing a system, progression toward a collective goal and preserving the balance of the system is the main goal.

### 5.3. Committed minorities

Although the collective behavior of units in the system is significantly important, the effect of single units or committed minorities is of particular interest. In the story, the Hoopoe is the guide that, encourages and advises each bird on its search to attain the ultimate Simorgh. In an experimental study (Couzin, 2011), it became evident that a committed minority of fish can overtop the consensus of a significantly larger school of fish, reestablishing mutability of opinions within the system.

Using decision making model, the effects of committed minorities were studied (Turalska, West, & Grigolini, 2013), where the authors tried to address the active role of committed groups in the Occupy Wall Street and the Arab Spring movements that started political changes of significant importance. In the brain, another study (Wallis, Anderson, & Miller, 2001) showed that single neurons in the prefrontal cortex are the neural basis of encoding abstract rules. By training two monkeys to use two abstract rules, the encoding of abstract rules by single neurons became evident.

Furthermore, a single neuron can take part in several networks, and depending on the state of the system, a single anatomical network can intercede in multiple functions (Kumar, 2010). By concurrent modulation of synaptic and cellular properties of its multiple target neurons, complete reorganization of the functional interactions among these cells can be quickly induced by the activation of a single neuromodulatory neuron. This activity will result in sudden dissolution of the precedent functional network driving a given motor pattern and controlling a different motor pattern in the emergence of a functional network. However, it is worth mentioning that the effect applies when the ambiguity concerning a given issue in the system exists, and it does not occur in all systems or any situation.

### 5.4. The mirror position

The mirror position used by Attār to express the Simorgh’s image in the lake has striking scientific implications. It enables the recognition of the intrinsic nature of the self, by comparing itself to a similar existing creature within the internal actions and reactions in the mirror. Mutual reactions lead to a complex chain of reactions, a better self-conception and clearer understanding of the world around. Having said that, Attār’s allusion to the mirror reminds the behavioral technique introduced by psychologist Gordon Gallup Jr, in 1970, known as Mirror Self-Recognition (MSR) test (Gallup, 1970).

MSR was assumed to be the hallmark of higher intelligence in humans and was used to assess whether a non-human animal encompasses the ability of self-recognition. It was shown that by training with visual-somatosensory association in Rhesus monkeys, they could obtain mirror-induced self-directed behaviors analogous to mirror self-recognition (Toda & Platt, 2015). Confrontation with the mirror caused the animal to become aware of the unseen self. On the other hand, the mirror represents the importance of measurement in systems where infinite probabilities collapse to a unique existence.

Observation of reflective behavior of another bird can serve as a mirror. In the story, mutual communication among birds and with the whole population of birds reminds us of the famous birdsong experiment (Mindlin & Laji, 2005; Garrod & Pickering, 2009). Also, in a recent study (Friston & Firth, 2015) the authors investigated the mutual interaction of two birds’ song. Based on their observations, they claimed that the notion of self originates in behavior and observation of behavior leads to a new version of self; repetition of the process leads to a new mind which embodies the notion of two communicating subjects. First, one may infer to mirror neurons that fire both when an animal is in action and when the animal perceives the same action performed by another as though the observer is acting itself (Rizzolatti & Craighero, 2004; Keysers, 2010).

Behavioral synchrony adjusts our minds for reasoning about minds in the process of developing social consonance and synergy. In other words, as a bird makes a sound, it touches the universe as if humanly speaks about the philosophy of life; he touches the reality of existence and by getting a feedback from the audience, refines his notions and understanding of life (Figure 2b).

In the story, however, bilateral observation of each bird throughout the story forms an infinite number of reflections representing the zenith of human experience through understanding and interactive calibration in the context of cooperative action and dialogue (Friston & Firth, 2015). Hence, the dialogue is among a population of birds from and with different backgrounds and beliefs, so that the feedback of each individual bird is shaped in more than a single bilateral communication, and a more refined version of this notion leads to a global understanding that we call consciousness.

### 5.5. Number 30

**Figure 3.**
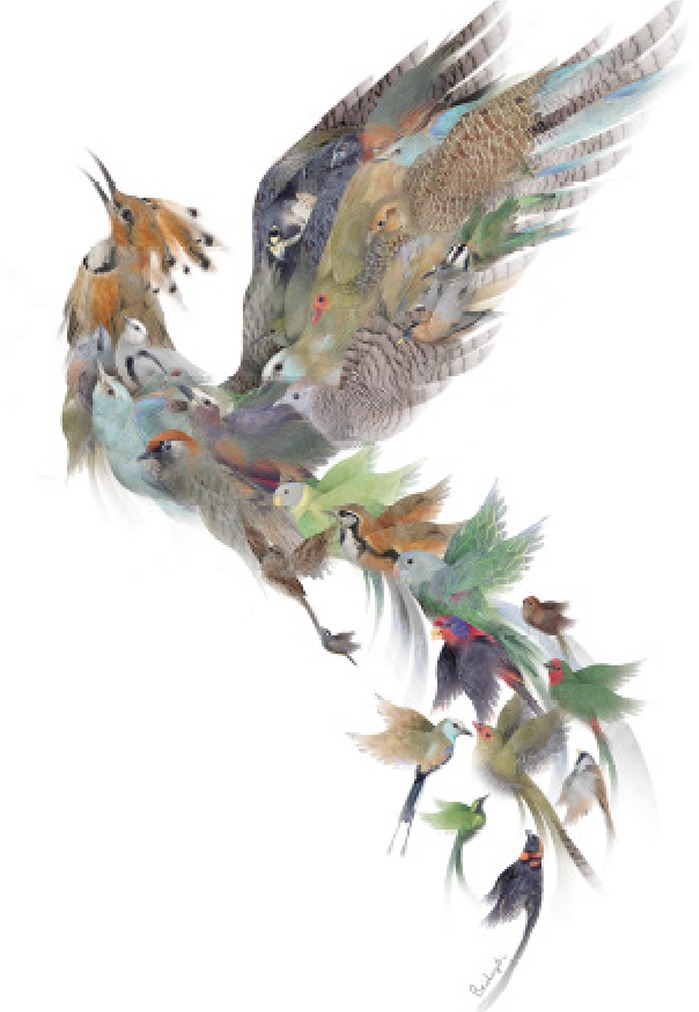
Simorgh sketched by Hamidreza Bidaghi, Fajr Film Festival, Tehran, Iran (The picture is provided by the artist)

Another feature of the story is the number of birds remaining as they reach the final destination of the journey (Figure 3). Studies show starlings are able to retain unison as a group in uncertain environments. The results suggest the evolution of flocking for starlings originates from the ability to sustain uncertainty (Young, Scardovi, Cavagna, Giardina, & Leonard, 2013). Although due to the exigency of the rhyme of poem, the choice of number thirty seems to be completely random, there are doubts that Attār has chosen the number just accidently. Thirty, in Attār’s view, might be an acceptable number for the establishment of a wise society. Notably, the number 30 remains a mysterious number in statistical studies and central limit theory, and as a rule of thumb, it is the minimum number of subjects in a statistical population (Chakrapani, 2011).

## 6. Summary and Conclusion

*The Conference of the Birds* restates the enthusiasm of a group of birds who have a common goal: the ambition to find the mystic Simorgh. Under the leadership of Hoopoe, they launch a journey toward the dwelling place of Simorgh. However, through metaphor, Attār alludes to the search for the meaning of the self through a captivating story that encompasses philosophical and scientific ideas. The search for self through a long and arduous journey by birds is comparable to a painful but beautiful human journey toward understanding. Even though the mystic bird, Simorgh was not what they expected it to be, they found what they were looking for as the quality of their existence and identity as Attār expresses somewhere himself: *Your meaning is composed of all of your understandings*.

Other poets have replicated the philosophical concerns of Attār’s poem in their own work. The poet Rumi was inspired by Attār’s ideas, and he interpreted Attār’s allusions in his own work. He seems to have had a unique insight into Attār’s articulation and metaphors. Quatrains in which explore the meaning and philosophical identity of humanity could be the sequel to the Simorgh story. *You are what you seek*.

The demeanor of animal groups such as swarms of insects, schools of fish or flocks of birds corresponds to the concept of collective mind. Throughout the story, besides explaining how a social complex network works together, posing an unprecedented challenge to the sense of who we are, Attār is unintentionally suggesting how a conference of birds resembles the way our brain works. In other thoughtful interpretation (Anvar & Twitty 2008), Rumi states that *A drop is an ocean only when it is in the ocean; otherwise, a drop is a drop and an ocean is an ocean.*

Now, despite the complexity of the world and its laborious social, political and economic situations, and over the centuries of tremendous human development in science and philosophy, the question is whether the man is capable of changing and controlling the overwhelming processes that threaten civilization and the nature of life.

We may deduce from the story that when we as humans reach self-awareness “a global consensus about a self-image of our civilization” and learn the true nature of life and the evolutionary changes from our past, as a part of life processes on the earth, to what we are now, when we appreciate the formation of cultures, rules, frameworks, religions and beliefs, and when we can perceive each individual entity in unison with all units, this would be a turning point in our history. When that happens, we can resolve many problems due to conflicts in our notions of the essence of the mind and life.

Strikingly, Simorgh story deserves to be the touchstone for questions on the nature of the mind, including the profound question of humanity’s search for the meaning of life as depicted in Attār’s captivating story Simorgh, the benevolent mythical flying creature, possesses the potency to answer these questions and concerns that are now the center of attention for cognitive scientists in topics including consciousness, self-awareness, and the brain and the mind. The story, though archaic, still speaks to us beautifully in allegorical verses and in a virtual dialogue.

## Ethical Considerations

### Compliance with ethical guidelines

No empirical experiment was conducted to write this paper.
